# Multiple Classifiers Based Semi-Supervised Polarimetric SAR Image Classification Method

**DOI:** 10.3390/s21093006

**Published:** 2021-04-25

**Authors:** Lekun Zhu, Xiaoshuang Ma, Penghai Wu, Jiangong Xu

**Affiliations:** 1 School of Resources and Environmental Engineering/Anhui Province Key Laboratory of Wetland Ecosystem Protection and Restoration, Anhui University, Hefei 230601, China; x18301080@stu.ahu.edu.cn (L.Z.); wuph@ahu.edu.cn (P.W.); x19201019@stu.ahu.edu.cn (J.X.); 2 Department of Resource and Environmental Sciences, Wuhan University, Wuhan 430072, China; 3 Information Materials and Intelligent Sensing Laboratory of Anhui Province, Anhui University, Hefei 230601, China

**Keywords:** polarimetric synthetic aperture radar, deep learning, majority voting, CV-CNN

## Abstract

Polarimetric synthetic aperture radar (PolSAR) image classification has played an important role in PolSAR data application. Deep learning has achieved great success in PolSAR image classification over the past years. However, when the labeled training dataset is insufficient, the classification results are usually unsatisfactory. Furthermore, the deep learning approach is based on hierarchical features, which is an approach that cannot take full advantage of the scattering characteristics in PolSAR data. Hence, it is worthwhile to make full use of scattering characteristics to obtain a high classification accuracy based on limited labeled samples. In this paper, we propose a novel semi-supervised classification method for PolSAR images, which combines the deep learning technique with the traditional scattering trait-based classifiers. Firstly, based on only a small number of training samples, the classification results of the Wishart classifier, support vector machine (SVM) classifier, and a complex-valued convolutional neural network (CV-CNN) are used to conduct majority voting, thus generating a strong dataset and a weak dataset. The strong training set are then used as pseudo-labels to reclassify the weak dataset by CV-CNN. The final classification results are obtained by combining the strong training set and the reclassification results. Experiments on two real PolSAR images on agricultural and forest areas indicate that, in most cases, significant improvements can be achieved with the proposed method, compared to the base classifiers, and the improvement is approximately 3–5%. When the number of labeled samples was small, the superiority of the proposed method is even more apparent. The improvement for built-up areas or infrastructure objects is not as significant as forests.

## 1. Introduction

Synthetic aperture radar (SAR) systems are active microwave imaging systems, which can obtain high-resolution images in both daytime and nighttime, and under all weather conditions [1]. As an advanced form of SAR, polarimetric SAR (PolSAR) systems can work in different polarization modes to characterize the observed land-cover types, and thus have a strong ability to obtain scattering information, resulting in their widespread use. As a result, PolSAR image classification has played an important role in many fields [2,3,4,5,6,7,8].

In the last three decades, a large number of algorithms have been developed for PolSAR image classification [9,10,11,12,13,14,15,16,17,18,19]. The traditional classifiers can be divided into three major categories. The first type is based on the statistical characteristics of PolSAR data. For example, Kong et al. [9] derived a maximum likelihood (ML) classifier for single-look complex (SLC) PolSAR imagery, which is based on a complex Gaussian distribution; for multilook PolSAR data, Lee et al. [10] applied the principle of ML and derived a Wishart distance measure; Liu et al. [11] proposed a superpixel-based classification method with an adaptive number of classes, which can take the spatial relations between pixels into consideration and make full use of the inherent statistical characteristics of PolSAR data; and Xie et al. [12] proposed a clustering-Wishart-auto-encoder classification model, which simultaneously considers the compactness and statistical distribution of the data by combining the k-means clustering algorithm with the objective function of a prior model. The second approach is focused on the inherent characteristics of the polarimetric scattering mechanisms and can take full advantage of the prior information about class type recognition. For example, Freeman et al. [13] proposed a hierarchical classification approach based on the scattering characteristics; based on the maximum entropy method, Kouskoulas et al. [14] proposed the Bayesian hierarchical classifier to classify low vegetation; Cheng et al. [15] proposed an unsupervised classifier to classify PolSAR pixels into eight combinations using three metrics extracted from the observed coherency matrix; and based on Freeman decomposition and H/Alpha decomposition, Zhao et al. [16] proposed a framework for the iterative classification of PolSAR data, which classifies the data into nine initial classes and obtains the final classification result through the use of a Wishart classifier. The third strategy uses a combination of polarimetric scattering characteristics and statistical properties. For example, Lee et al. [17] proposed an approach which uses both H/α target decomposition [18] and the ML classifier based on a complex Wishart distribution; and Chang et al. [19] proposed a PolSAR image classification method based on the degree of polarization and co-polarized phase-difference statistics, which has the ability to classify PolSAR data into four major classes. Hao et al. [20] proposed a classification framework for PolSAR image, which combined XGBoost, superpixels generation, and majority voting.

In the last few years, deep learning-based classifiers have become more and more widely used in the field of PolSAR image classification [21,22,23,24,25,26,27,28,29]. As a subset of machine learning, deep learning can process complex data efficiently, and has a strong feature extraction capability. For example, Hou et al. [21] applied a multilayer autoencoder and super-pixels for PolSAR image classification, which can make good use of the scattering characteristics and spatial relationships between pixels; and Liu et al. [22] utilized a task-oriented generative adversarial network (GAN) for PolSAR image classification and clustering. Li et al. [23] used the Deeplabv3 to segment fruit and twigs from the background based on the color and depth information in a RGB picture. Among the deep discriminative networks, the real-valued convolutional neural network (RV-CNN) model is one of the most popular models [25]. On account of the special multidimensional convolution operations, the RV-CNN model has significant advantages in image data classification [26]. However, the RV-CNN model only uses the amplitude of the PolSAR imagery, while neglecting the phase information. For PolSAR data represented by a covariance or coherency matrix, the phase of off-diagonal elements plays an important role in classifying different types of scatterers. Hirose et al. [27] first processed complex-valued data by a complex-valued neural network, in which weight and neural activation functions are all complex-valued. Soon afterwards, a novel complex-valued CNN (CV-CNN) classification method was proposed by Zhang et al. [28], which not only takes the amplitude and phase information of the PolSAR imagery as input, but also propagates the phase information through all the processes of the network.

Deep learning has achieved enormous success in PolSAR data interpretation [30], and generally obtains better performances than the traditional classifiers. However, the classification result of a deep learning network is dependent, to a large extent, on a large number of labeled samples [31,32]. Limited training samples can lead to an overfitting phenomenon, which refers to the fact that the model only learns the characteristics of the training samples in the training process, and the classification result on the test set is poor because of the low generalization performance of the trained model. The lack of labeled samples can lead to a poor classification result. Therefore, semi-supervised classification has become prevalent in deep learning because it avoids the labor-intensive task of acquiring a large number of labeled samples [33,34], and it can fully exploit the available labeled training samples. In the field of optical image classification, many semi-supervised algorithms have been developed in the past few decades [35,36,37]. However, the semi-supervised algorithms for PolSAR data classification are relatively deficient. In order to solve the lack of labeled datasets and minimize the speckle noise, Li et al. [38] proposed a semi-supervised algorithm based on self-training and superpixel, which used the segmentation and stacked sparse auto-encoder to expand the training set. Sun et al. [39] proposed an impartial semi-supervised learning strategy based on extreme gradient boosting to classify very high-resolution images with imbalanced data. Liu et al. [40] proposed a novel semi-supervised algorithm with neighborhood constraints to reduce the cost of labeled samples, which uses a number of PolSAR features of every pixel, including its neighbors, to construct a spatial group; and Hou et al. [41] proposed a robust semi-supervised probability graphic-based classification framework to solve the problem of the weak quantity and quality of the labeled training samples.

Another weakness of deep learning is that it may have difficulties in discriminating objects with similar textures, but with different scattering patterns [42]. In contrast, the traditional classifiers, such as the Wishart classifier, are based on a complex Wishart distribution, and can employ the scattering traits of PolSAR data. Hence, every method has its own advantages. Fusion strategies based on multiple classifiers have been developed, the main purpose of which is to make full use of the respective advantages of each classifier [43]. Theoretically, the combination of multiple classifiers is generally able to obtain a better classification result [44]. Classification methods can be integrated according to the way in which they are built. Majority voting is one of the most widely used multiple-classifier integration strategies, which involves selecting the predicted class with the most votes [45].

In the proposed method, motivated by the above viewpoints, we combine the deep learning technique with the traditional classifiers, the aim of which is to take advantage of each classifier and improve the overall accuracy (OA) of classification, even in the case of limited training samples. Firstly, the classification results of the Wishart classifier, SVM classifier, and CV-CNN model are used to conduct majority voting. By doing so, the labeled training samples are expanded by dividing the classification result into a strong dataset and a weak dataset. The strong dataset are used as pseudo-labels to reclassify the weak dataset by CV-CNN. In order to make full use of the strong dataset, the weak dataset is reclassified three times using the pseudo-labels derived by the strong dataset, and then followed by a majority voting manner to integrate the three classification results.

The main contributions of this present paper are as follows: A novel sample selected method is maintained by a voting strategy, which is used to expand labeled training samples based on three classifiers classification results; a novel semi-supervised classification method is presented, which can make full use of scattering characteristics and obtain a high classification accuracy based on limited labeled samples.

The rest of this paper is organized as follows. The characteristics of PolSAR data and the traditional classifiers are described in Section 2. In Section 3, the CV-CNN model and the proposed method are introduced. The experimental results obtained with the benchmark PolSAR images are described in Section 4. Finally, our conclusions are drawn in Section 5.

## 2. Related Works

### 2.1. PolSAR Data

Fully polarimetric SAR systems measure the complex scattering characteristics of an observed target with quad-polarizations. Each pixel of a PolSAR image in the SLC format can be expressed by a complex scattering matrix:

(1) $$S=\left[\begin{array}{cc} S_{hh} & S_{hv}\\ S_{vh} & S_{vv} \end{array}\right]$$

where each element in the complex scattering matrix represents the amplitude and phase information. *S_vh_* denotes the scattering coefficient of the horizontal transmitting and vertical receiving polarizations, which can be characterized by the phase $\phi_{vh}$ and the amplitude $\left|S_{vh}\right|:$$S_{vh}=\left|S_{vh}\right|e^{j\phi_{vh}}=\left|S_{vh}\right|\cos\phi_{vh}+j\left|S_{vh}\right|\sin\phi_{vh}$, where *j* is the imaginary unit. The other elements are similarly defined. Under a monostatic case and the reciprocity theorem, *S_hv_* and *S_vh_* are equivalent. Thus, under Pauli decomposition [46], the complex scattering vector *h* can be obtained as:

(2) $$h=\frac{1}{\sqrt{2}}{\left[S_{hh}^{}+S_{vv}^{},S_{hv}^{}+S_{vv}^{},2S_{hv}^{}\right]}^{T}$$

where the superscript *T* is the transpose operation. The coherency matrix of the PolSAR imagery for the multilook case can be expressed as:

(3) $$T=\frac{1}{n}\sum_{i=1}^{n}h_{i}h_{i}{}^{H}=\left[\begin{array}{ccc} T_{11} & T_{12} & T_{13}\\ T_{21} & T_{22} & T_{23}\\ T_{31} & T_{32} & T_{33} \end{array}\right]$$

where *n* is the number of looks, and the superscript *H* represents the conjugate transpose.

### 2.2. Traditional Classifiers

For the traditional classifiers applied in PolSAR image classification, Wishart and SVM are the classical methods. Wishart and SVM are both supervised classifiers which play an important role in the field of PolSAR data interpretation.

#### 2.2.1. Wishart Classification

For multilook PolSAR data represented in coherency matrix *T*, Lee et al. [10] applied the principle of ML and derived a Wishart distance:

(4) $$d\left(T,V_{m}\right)=Tr\left(V_{m}^{-1}T\right)+\ln\left|V_{m}\right|$$

where *d*(*T*, *V_m_*) means a distance between the sample pixel covariance matrix *T* and cluster center *V_m_*. For each target pixel, the Wishart distance between it and each cluster center is calculated, and the pixel is classified to the class with the smallest Wishart distance. The Wishart distance is a measurement that is commonly used to measure the similarity of PolSAR imagery. For example, Jiao et al. proposed a Wishart deep stacking network method to improve the precision of PolSAR image classification, based on the rapid implementation of the Wishart distance [47].

#### 2.2.2. Support Vector Machine Classification

SVM is a classification algorithm which minimizes the structural risk to improve the generalization ability of machine learning. SVM also minimizes the empirical risk and confidence range to obtain good classification results, even in the case of limited labeled samples [48]. Due to the superiority of SVM, a lot of algorithms have been developed based on SVM. For example, Maghsoudi et al. [49] proposed a system consisting of a feature selector based on a non-parametric evaluation function and SVM; SVM was combined with a radial basis kernel function and stochastic distance to assess the robustness of in region-based classification by Negri et al. [50].

#### 2.2.3. Deep Learning Method for PolSAR

Deep Learning has demonstrated great advantages in the PolSAR image processing task. The deep learning method is a series of algorithms based on a complex structure, using nonlinear transformation to abstract the multiple high-level characteristics, whose essence is acquiring high-level characteristics by a multi-layer network. This is based on a large amount of data, so as to eventually improve the accuracy of classification and provide a new idea for high-level characteristics. Auto encoders (AE), deep belief network (DBN), and CNN are the main methods that have been widely used in PolSAR image application. PolSAR image classification based on the deep learning method generally consists of image preprocessing, dataset production, parameter testing, and result analysis.

**Figure 1:**
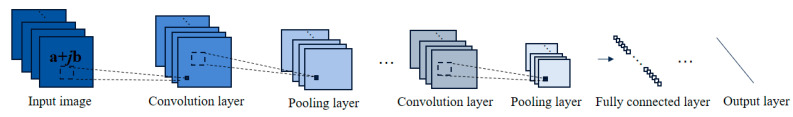
Framework of the CV-CNN model.

Deep learning has achieved great success in the field of image classification [51], and the CV-CNN model can be considered as a representative method. As shown in Figure 1, as in the traditional RV-CNN model, the CV-CNN model consists of an input layer, an output layer, convolutional layers, pooling layers, and fully connected layers [28]. However, all elements of the CV-CNN model are complex-valued, including the input layer, convolutional layer, activation function, pooling layer, and output layer. Compared with the RV-CNN model, there are three main advantages of the CV-CNN model [28]: (1) the CV-CNN model not only uses the amplitude of the PolSAR imagery, but it also propagates the phase information through all the process; (2) all the mathematical operations of the whole network are extended under complex analysis theory, and at the same time, both the data and parameters are extended into the complex field; and (3) a complex back-propagation algorithm based on stochastic gradient descent is used for the model training. In the following, the details of the CV-CNN model are presented.

The function of the convolutional layer is to convolve the filters to extract the different features of the previous layer. For the input image, each filter bank will detect specific regional features, which means that every feature map represents a specific feature at different zones of the previous layer. The output of the (*l* + 1)th convolutional layer can be represented as:

(5) $$O_{m}^{\left(l+1\right)}=f(\xi (V_{m}^{\left(l+1\right)}))+jf(\psi (V_{m}^{\left(l+1\right)}))$$

(6) $$p^{\left(l+1\right)}=\sum_{k}^{K}w_{pK}^{\left(l+1\right)}*O_{k}^{\left(l\right)}+b_{p}^{\left(l+1\right)}$$

where * represents the convolution operation. ξ and ψ are, respectively, the real and imaginary parts of a complex-valued domain. $V_{m}^{\left(l+1\right)}$ is the *m*th output feature map of the (*l* + 1)th layer. $w_{pK}^{\left(l+1\right)}$ denotes the filter banks. $O_{k}^{\left(l\right)}$ and $b_{p}^{\left(l+1\right)}$ represent the previous layer’s input feature maps and the bias, respectively. f(⋅) is the nonlinear function, which represents the sigmoid function.

The pooling layer usually follows the convolutional layer, which can not only simplify the spatial structure, but can also merge similar features of the input feature maps. Among the many pooling functions, the max pooling and average pooling layers are the two most commonly used functions. Hence, pooling layers can be regarded as downsampling layers.

In general, one or more fully connected layers are employed in the CV-CNN model, and can be regarded as special convolutional layers. In addition, each neuron of the fully connected layer is connected to all the neurons in the front layer. The output can be expressed as:

(7) $$O_{n}^{\left(l+1\right)}=f(\xi (V_{n}^{\left(l+1\right)})+jf(\psi (V_{n}^{\left(l+1\right)}))$$

(8) $$V_{p}^{\left(l+1\right)}=\sum_{m=1}^{M}w_{pm}^{\left(l+1\right)}{\cdot O}_{m}^{\left(l\right)}+b_{p}^{\left(l+1\right)}$$

where *M* denotes the quantity of neurons at the *l*th fully connected layer.

The final output layer is a classifier represented in a 1 * *C* complex-valued vector, which indicates the probability of the pixel belonging to the *C*th class. After this, all of the parameters in the network are learned in a supervised way by minimizing a loss function *E*, which can be written as:

(9) $$E=\frac{1}{2}\frac{1}{N}\sum_{n=1}^{N}\sum_{k=1}^{K}[(\xi (T_{k}[n])-\psi (O_{k}[n]){)}^{2}-{\left(\psi (T_{k}[n])-\psi (O_{k}[n])\right)}^{2}]$$

where T[n] is the *n*th input data. The covariance matrix *T* is a Hermitian matrix. Hence, we only take the upper triangular elements of the matrix as the input data.

## 3. The Proposed Approach

### 3.1. The Deep Learning Method

Deep Learning has shown great advantages in PolSAR image processing task. The deep learning method is a series of algorithms based on a complex structure using nonlinear transformation to abstract the multiple high-level characteristics, whose essence is acquiring high-level characteristics by a multi-layer network. This is based on a large amount of data, so as to eventually improve the accuracy of classification and provide a new idea for high-level characteristics. Auto encoders (AE), deep belief network (DBN), and RV-CNN are the main methods that have been widely used in PolSAR image application.

The CV-CNN model, as an advanced form of RV-CNN, has achieved great performances in PolSAR image supervised classification. However, its classification performance is heavily reliant on the number of labeled training samples, and limited training samples can lead to an overfitting phenomenon, resulting in the low generalization performance of the trained model. The CV-CNN model may have difficulties in discriminating objects with similar textures, but with different scattering patterns. On the contrary, the Wishart classifier can employ the scattering traits of PolSAR data, but the spatial information in the image has not been fully utilized. Generally, the single classifier is unable to accurately abstract high-level description of ground objects. Hence, discovering a method that can take the scattering traits and the multiple high-level characteristics deserves extensive research.

In this paper, with the aim of solving the problem of insufficient labeled samples, a novel supervised classification method is proposed. The main idea is to take advantage of each classifier, and expand the number of high-reliability samples, so as to improve the classification accuracy of the CV-CNN model.

### 3.2. Configuration of the Proposed Method

**Figure 2:**
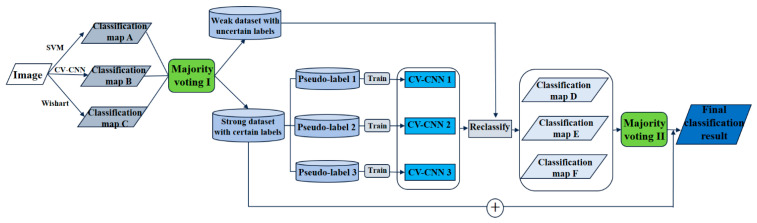
Framework of the proposed model.

As illustrated in Figure 2, the flowchart of the proposed method consists of two main parts. Firstly, a small number of labeled samples are randomly selected to classify the PolSAR images by three base classification methods, which are SVM, Wishart, and CV-CNN. A majority voting strategy is then employed to fuse the base classifier results, which is done by expanding the labeled training samples by dividing the classification result into a strong dataset and a weak dataset. In this majority voting process, if the three classifiers are unanimous in the class of a certain pixel, this pixel is categorized into the strong dataset with the voted class label; otherwise, it is categorized into the weak dataset with the uncertain class label.

After acquiring the strong dataset and expanding the number of training samples, more samples could, in theory, be employed to classify the PolSAR data. In general, it is easier and faster to classify the weak dataset only once by the CV-CNN model. In order to make full use of the strong dataset and suppress the interference of misclassified pixels in the initial majority voting process, samples are selected three times from the strong dataset as pseudo-labels to train the CV-CNN classifier three times, followed by normal majority voting, which is based on the predicted class with the most votes. Finally, by combining the strong dataset and the reclassification result of the weak dataset, the final classification results are obtained.

### 3.3. Preprocessing of PolSAR Data for CV-CNN

In order to speed up the gradient descent and improve the classification accuracy, it is necessary to preprocess the PolSAR data. For each element of the coherency matrix of the input data, the average *T*_ave_ and the standard deviation *T*_std_ of the training samples are calculated. For the off-diagonal terms, for example *T*_12_, the average and standard deviation can be calculated as:

(10) $$T_{12\_\mathrm{ave}}=\frac{1}{K}\sum_{k=1}^{K}T_{12}\left(k\right)$$

(11) $$T_{12\_\mathrm{std}}=\sqrt{\frac{\sum_{k=1}^{K}\left(T_{12}\left(k\right)-T_{12\_\mathrm{ave}}\right)\left(\bar{T_{12}\left(k\right)-T_{12\_\mathrm{ave}}}\right)}{K}}$$

and the final normalized data are obtained as:

(12) $$T^{\prime }{}_{12}=\frac{T_{12}-T_{12\_\mathrm{ave}}}{T_{12\_\mathrm{std}}}$$

The diagonal terms are similarly defined.

## 4. Experimental Section

In this section, we describe the experiments on two real PolSAR images to demonstrate the performance of the proposed algorithm (Table 1). The OA and Kappa were adopted to quantitatively evaluate the performance of the different methods. The refined Lee filter [52] was applied to reduce the speckle noise before conducting the experiments. For SVM classification on PolSAR data, selecting proper parameter settings and the kernel parameter was an important step. In this study, we selected all the terms of the coherency matrix as the input polarimetric indicators and chose the Gaussian radial basis function (RBF) as the kernel function, which was implemented in PolSARpro V6.0 software. All of the experiments were implemented in the MATLAB R2018a environment on a 3.2 GHz machine with GTX 1060 GPU and 16-GB memory. The CV-CNN model consists of an input layer, an output layer, two convolutional layers and one pooling layer. The size of the input layer was 12×12×6, which means that the channel number was 6, and the local patch was 12×12. Then, the input data was filtered by six convolution filters with the size of 3×3×6, producing six feature maps of size 10×10. The size of average pooling layer was 2×2 and stride was 1. The size of the second convolutional layer filter was 3×3×6×12, resulting in 12 feature maps and 3×3 size. The fully connected layer consisted of 108 neurons. The output layer contained *q* neurons and q is the number of classification classes.

### 4.1. Experiments with the Flevoland Dataset

**Figure 3:**
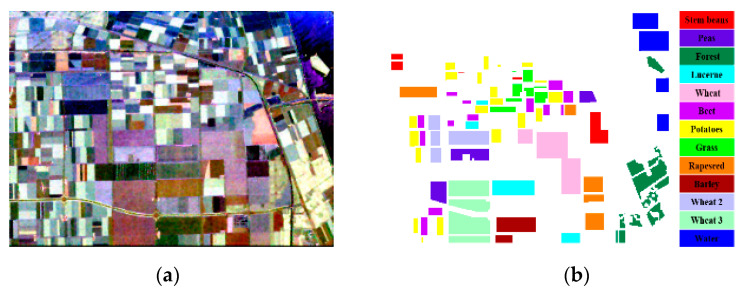
Original Flevoland dataset. (**a**) Pauli RGB image for the Flevoland dataset. (**b**) Ground truth and legend of (**a**).

The first dataset used for the verification of the proposed method was acquired by the NASA/JPL AIRSAR platform in 1989, over an agricultural area in Flevoland in the Netherlands. The size of the image is 750 × 1024 pixels. Figure 3a displays the Pauli RGB image of the data, which was formed by the intensities of the Pauli de-composition. The ground truth and the corresponding legend are shown in Figure 3b. In total, there are 13 classes, including stem beans, peas, forest, lucerne, beet, potatoes, grass, rapeseed, barley, water, and three types of wheat.

**Figure 4:**
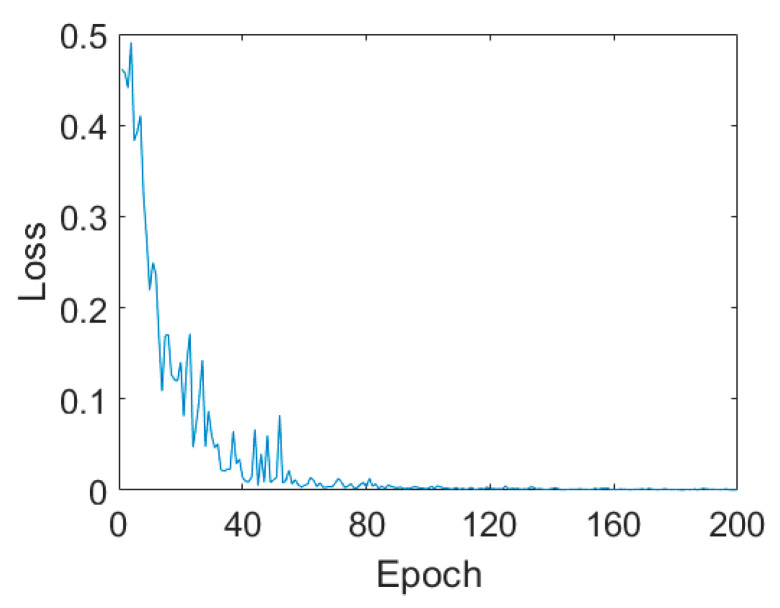
Loss curves on the Flevoland dataset.

In this experiment, we randomly selected only 509 labeled samples. That is to say, only 39 pixels were taken as the training samples for each class. The training sample ratio was only 0.34%, which is quite a low level when compared with other studies. The classification results are shown in Figure 3 and the quantitative assessment results are listed in Table 2. For the first CV-CNN experiment on Flevoland dataset, the hyperparameters were set as follows. The learning rate was 0.6, and the batch size was 10 with 200 epochs. The loss curves are shown in Figure 4. From the loss curves, we can easily find that with the increasing of epoch, the loss decreased quickly. When the epoch is 100, the loss was stable at 0.006.

**Figure 5:**
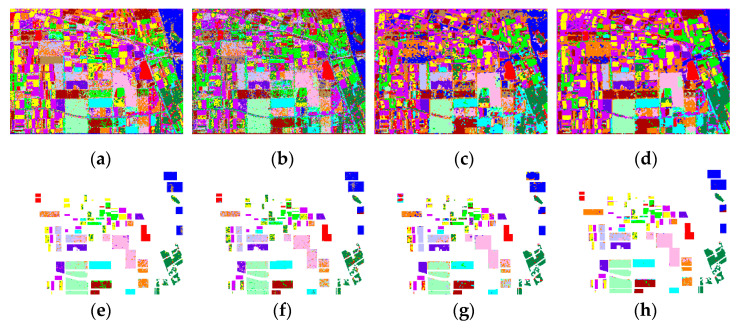
Classification results for the Flevoland dataset. (**a**–**d**) Classification results of the different approaches: (**a**) Wishart, (**b**) SVM, (**c**) CV-CNN, (**d**) proposed method. (**e**–**h**) Results overlaid with the ground-truth maps of (**a**–**d**), respectively.

From Table 2 we can see that the OAs of the Wishart, SVM, and CV-CNN base classifiers, the strong dataset, and the proposed method, were 87.04%, 80.78%, 81.77%, 97.34%, and 90.75%, respectively. The highest OA was achieved by the proposed method, and the classification accuracies for all categories with the proposed method are higher than those with CV-CNN. It can also be seen that the accuracy for every class with the strong dataset was higher than for the base classifiers. The OA for the strong dataset was higher than for the base classifiers, which validates the high reliability of the strong dataset. On one hand, majority voting can, on the whole, improve the accuracy. On the other hand, the OA for the strong dataset was very high, so this dataset can be taken as pseudo-labels to reclassify the weak dataset. As shown in Table 2, the number of training samples of stem beans was only 41, however, the number of samples of stem beans in the strong dataset was 10,934 with a high accuracy of 99.98%, which indicates that the labeled training samples were expanded by the proposed method. The low classification accuracy of CV-CNN can be predominantly attributed to the low accuracy obtained for the rapeseed class, while the low accuracy of SVM can be attributed to the low accuracy obtained for the potatoes class and rapeseed class. This directly degrades the performance of the majority voting approach, thus leading to the poor performance of the proposed method in classifying the rapeseed and potato classes. There are also many fragments in the classification results of Wishart, SVM, and the proposed method, as shown in Figure 5e–g, respectively. From Figure 5c, it is clear that the misclassification is severe in the right side and top of the Flevoland image classified by CV-CNN. In addition, the results of the proposed method in Figure 5h appear much smoother than the results of the other methods. This indicates that the proposed method can maintain a good classification result, even with a small number of labeled samples.

**Figure 6:**
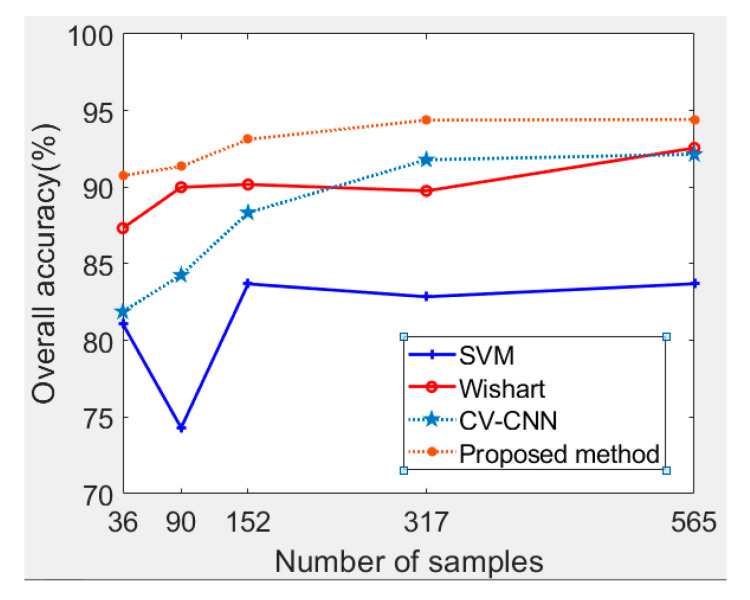
Trends of the OA with different numbers of training samples for the Flevoland dataset.

To further analyze the improvement of the proposed method, a series of experiments were conducted. For the Flevoland dataset, the trends of accuracy with the increase of the training samples are shown in Figure 6. It can be seen that, with the increase in the number of training samples, the OA of each method generally increased. The superiority of the proposed method was more significant when the number of samples was small, especially when compared with CV-CNN. The accuracy of SVM was relatively poor, and its accuracy fluctuates with the increase of the samples. With 90 training samples in particular, the classification accuracy was only 74.3%. In general, the Wishart classifier was stable with different numbers of samples, and the OA is around 88.0%.

### 4.2. Experiments with the Oberpfaffenhofen Dataset

**Figure 7:**
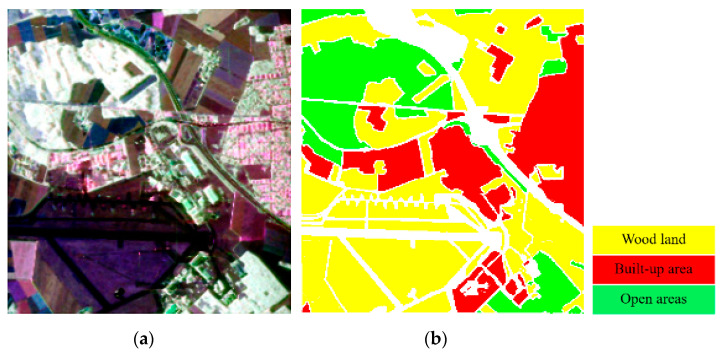
Original Oberpfaffenhofen dataset. (**a**) Pauli RGB image. (**b**) Ground truth and legend of (**a**).

The second experiment was carried out with the dataset obtained over the German Aerospace Center at Oberpfaffenhofen in Germany. This image was acquired by the ESAR system. The Oberpfaffenhofen image, as a benchmark dataset, is widely used for PolSAR data classification research. According to the ground truth shown in Figure 7, there are three identified classes in the image, i.e., built-up areas, wood land, and open areas. The size of the image is 1300 × 1200.

**Figure 8:**
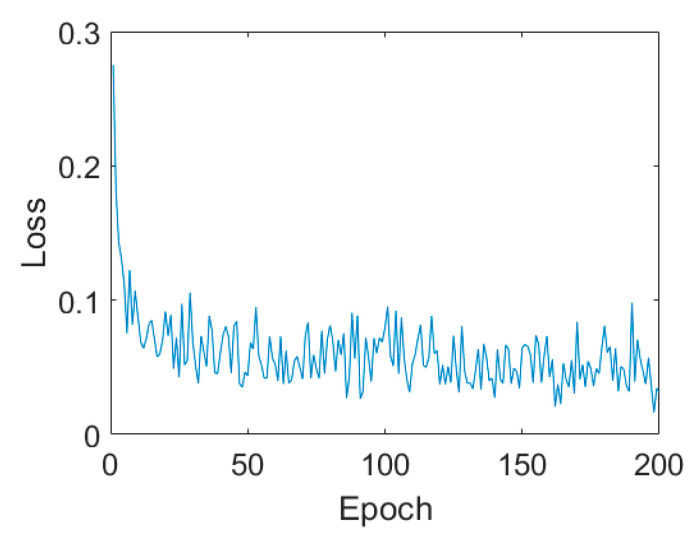
Loss curves on Oberpfaffenhofen dataset.

In this experiment, we selected only 446 labeled samples; in other words, only 149 pixels were taken as the training samples for each class. The training sample ratio was only 0.18%, which is quite a low ratio. For the first CV-CNN experiment, on Oberpfaffenhofen dataset, the hyperparameters were set as follows. The learning rate was 0.8, and the batch size was 10 with 200 epochs. The loss curves are shown in Figure 8. With the increasing of epoch, the loss fluctuated, but the tendency decreased.

**Figure 9:**
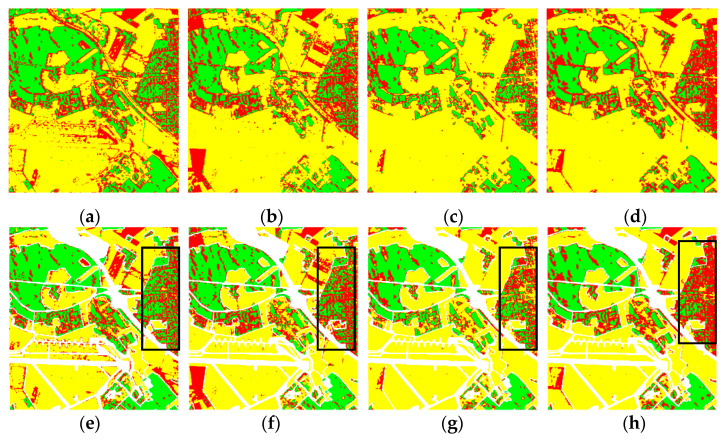
Classification results for the Oberpfaffenhofen dataset. (**a**–**d**) Classification results of the different approaches: (**a**) Wishart, (**b**) SVM, (**c**) CV-CNN, (**d**) proposed method. (**e**–**h**) Results overlaid with the ground-truth maps of (**a**–**d**), respectively.

The classification results are shown in Figure 9 and listed in Table 3. From Table 3, we can see that the OAs for the Wishart, SVM, and CV-CNN base classifiers, the strong dataset and the proposed method are 75.70%, 77.01%, 78.89%, 87.47%, and 82.76%, respectively. The highest OA was again achieved by the proposed method, and the classification accuracies obtained by the proposed method for most categories were higher than those ob-tained by CV-CNN, except for the open areas class. The classification accuracies obtained by the Wishart, SVM, and CV-CNN base classifiers for the built-up area class were all below 50%, thus leading to the poor performances of the strong dataset and the proposed method in classifying the built-up area class. The black rectangle in Figure 9 indicates the distinctly different results of the above classifiers. From Figure 9g, it can be seen that the misclassification in the built-up area class for CV-CNN is serious, especially in the right of the image, which is consistent with the data in Table 3. There are also too many fragments in the Wishart and SVM classification maps (Figure 9e,f). For the built-up area class, the accuracy of the proposed method shows a significant improvement over CV-CNN, thanks to the good classification result of the strong dataset. It is clearly demonstrated that, compared with base classifiers, the accuracy of the proposed method on every land-cover type shows a distinct improvement. In addition, the visual effect of the proposed method in the classification map is much smoother than the effect of the other methods.

**Figure 10:**
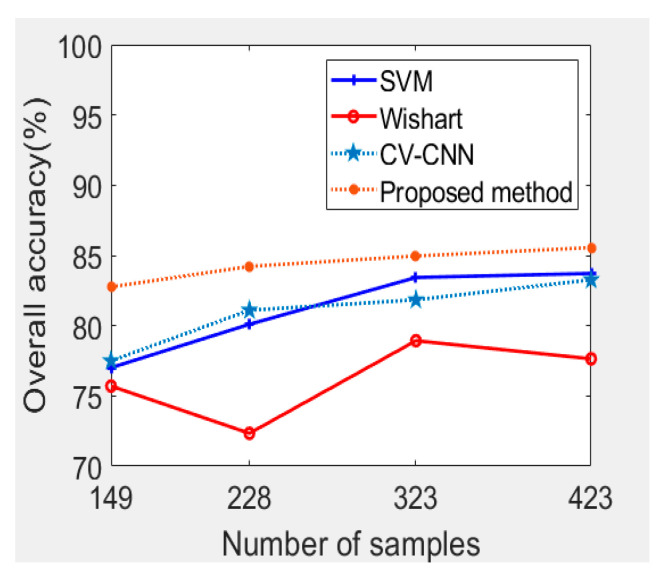
Trends of the OA with different numbers of training samples for the Oberpfaffenhofen dataset.

A series of experiments were carried out to further demonstrate the advantages of the proposed method. As shown in Figure 10, we can observe that, generally speaking, with the increase of the training samples, the OAs of each method show a gradual increase. The lowest OA was realized by the Wishart classifier. The Wishart classifier’s performance fluctuates with the increase of the training samples. The OA of SVM was about 82%, which is a similar performance to CV-CNN. The OA of the proposed method is always higher than that of the base classifiers, especially when the training samples are limited. At the right side of the Oberpfaffenhofen image, the buildings are sparsely distributed, and there are some forests, which makes the scattering characteristics similar to wood land. As shown in Table 3, the majority of accuracy in every land cover class was lower than open areas, which leads to a low accuracy of build-up area and wood land, and this leads to a small improvement in the Oberpfaffenhofen dataset experiment, compared to the Flevoland dataset experiment.

## 5. Conclusions

Deep learning methods have become more and more widely used in the field of PolSAR image classification. However, when the labeled training dataset is insufficient, the classification results are usually unsatisfactory. A novel semi-supervised classification method, which combines the deep learning technique with the traditional classifiers, has been proposed in this paper. The proposed method can take advantage of each classifier to generate a strong dataset and a weak dataset. The strong training set are used as pseudo-labels to reclassify the weak dataset by CV-CNN, which can make full use of the high-accuracy strong dataset. The final classification results are obtained by combining the strong training set and the reclassification results. The innovation of this method is that a majority voting approach is used to increase the training samples of the CV-CNN model, to further improve the classification performance. The experimental results obtained on two real PolSAR images confirmed the superiority of the proposed method with regard to the traditional supervised classifiers, especially when the number of original training samples was small. One of the limitations is that the settings of hyperparameters is slightly sophisticated in the proposed method and this makes it slightly time consuming. On one hand, our future work will focus on developing an automatic parameter optimization for the proposed method, and further investigation of the interactive connection between deep learning and the traditional scattering trait-based classifiers; on the other hand, our future work will carry out experiments on mountain areas to further verify the validity of the proposed method, and finally, space-borne PolSAR data also deserve to be researched.

## Figures and Tables

**Table 1:** Basic parameters of the dataset.

Data Set	Platform	Polarization	Spatial Resolution	Band	Number of Looks	Size
Flevoland	AIRSAR	Quad-polarization	10 m × 10 m	L	4	1024 × 750
Oberpfaffenhofen	ESAR	Quad-polarization	3 m × 3 m	L	1	1300 × 1200

**Table 2:** Classification precision for the Flevoland dataset (%).

Class	Number of Samples	Wishart	SVM	CV-CNN	Strong Dataset	Number of Samples in Strong Dataset	Proposed Method
Stem beans	41	99.87	97.28	81.91	99.98	10,934	97.72
Peas	61	89.31	83.23	97.07	99.22	12,288	97.93
Forest	45	89.94	86.17	91.96	99.01	20,614	96.83
Lucerne	38	97.70	94.54	90.72	99.98	15,469	96.82
Wheat	48	92.84	81.81	94.95	99.89	18,442	96.63
Beet	32	95.35	97.19	84.53	99.66	62,295	95.64
Potatoes	17	83.27	53.57	58.14	85.24	25,780	80.93
Grass	25	86.12	80.32	78.99	94.10	27,155	84.59
Rapeseed	33	65.82	50.39	49.24	78.45	12,927	84.41
Barley	17	78.73	88.90	89.99	99.84	19,934	91.29
Wheat 2	47	67.02	76.97	84.72	92.42	8520	82.64
Wheat 3	57	93.39	92.63	91.52	99.66	24,822	97.41
Water	48	90.78	88.95	87.12	99.58	19,653	96.19
OA	--	87.04	80.78	81.77	97.34	--	90.75
Kappa	--	85.96	79.18	80.25	97.11	--	89.96

**Table 3:** Classification precision for the Oberpfaffenhofen dataset (%).

Class	Wishart	SVM	CV-CNN	Strong Dataset	Proposed Method
Built-up area	43.66	46.24	36.86	61.28	55.46
Wood land	90.05	81.29	77.39	93.19	78.67
Open areas	85.16	89.28	98.06	99.89	96.29
OA	75.70	77.01	78.89	87.47	82.76
Kappa	61.48	62.46	63.41	75.21	71.47

## Data Availability

The data presented in this study are openly available in https://earth.esa.int/eogateway/.
